# [Corrigendum] Targeted silencing of CXCR4 inhibits epithelial-mesenchymal transition in oral squamous cell carcinoma

**DOI:** 10.3892/ol.2025.15427

**Published:** 2025-12-10

**Authors:** Yuansheng Duan, Shu Zhang, Longlong Wang, Xuan Zhou, Qinghua He, Su Liu, Kai Yue, Xudong Wang

Oncol Lett 12: 2055–2061, 2016; DOI: 10.3892/ol.2016.4838

Subsequently to the publication of the above article, an interested reader drew to the authors’ attention that, for the scratch-wound assay data shown in Fig. 1D, the data panels shown for the 0 h experiments (the Control, NC and si-CXCR4 panels) all appeared to show overlapping sections of data, suggesting that these data were derived from the same original source..

After having examined their original data, the authors have realized that these panels were inadvertently misused during the assembly of the figure, and that the data panel was shown correctly for the 0 h/Control experiment. The revised version of Fig. 2, now showing the correct data for the 0 h/NC and 0 h/si-CXCR4 panels, is shown on the next page. Note that the errors made in compiling this figure did not affect either the results or the conclusions reported in this paper, and all the authors agree to the publication of this Corrigendum. The authors thank the Editor of *Oncology Letters* for allowing them the opportunity to publish this Corrigendum, and apologize both to the Editor and to the readership of the Journal for any inconvenience caused.

## Figures and Tables

**Figure 1.**
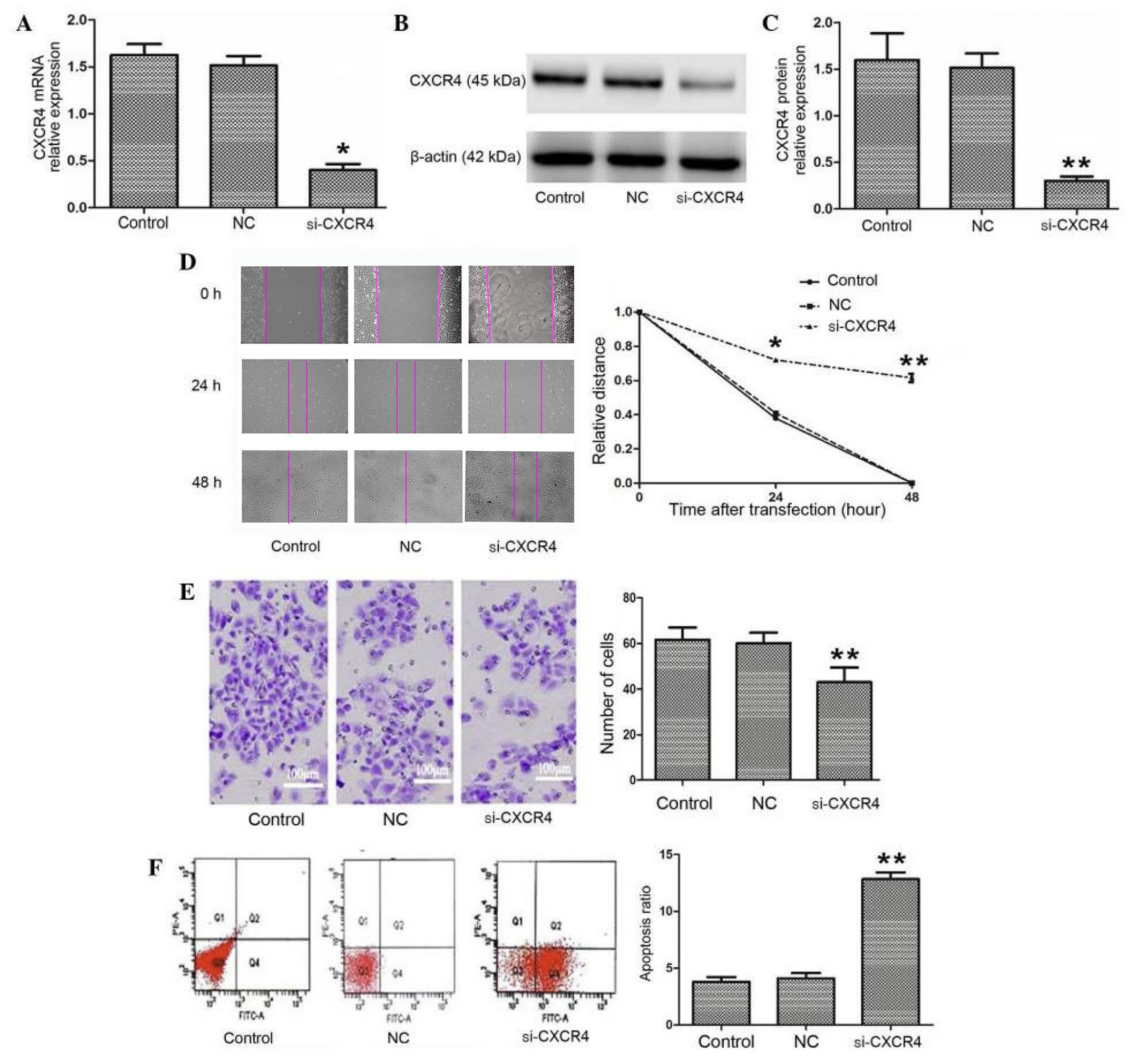
Silencing of CXCR4 inhibits invasion and migration and promotes apoptosis in TSCCA cells. (A-C) si-CXCR4 inhibited the expression of CXCR4 mRNA and protein. (D) A wound healing assay was performed to investigate TSCCA migration following transfection with si-CXCR4. si-CXCR4 significantly suppressed cells migration. (E) A Transwell assay was performed to compare the invasive capacities of TSCCA cells. si-CXCR4 inhibited cell invasion *in vitro* (magnification, ×400). (F) si-CXCR4 mediated an increase in the apoptosis of TSCCA cells. *P<0.05 and **P<0.01 vs. control. CXCR4, C-X-C chemokine receptor type 4; si-CXCR4, small interfering RNA to CXCR4; TSCCA, tongue squamous cell carcinoma; control, blank control group; NC, negative control small interfering RNA group.

